# Antioxidant Compound Extraction from Maqui (*Aristotelia chilensis [Mol]* Stuntz) Berries: Optimization by Response Surface Methodology

**DOI:** 10.3390/antiox6010010

**Published:** 2017-02-02

**Authors:** Issis Quispe-Fuentes, Antonio Vega-Gálvez, Víctor H. Campos-Requena

**Affiliations:** 1 Department of Food Engineering, University La Serena, Raúl Bitrán 1305, Box 599, La Serena, Chile; avegag@userena.cl; 2 Department of Polymers, Faculty of Chemical Sciences, University of Concepcion, Edmundo Larenas 129, Casilla 160-C, Concepción, Chile; vcamposr@udec.cl

**Keywords:** antioxidant activity, D-optimal design, Maqui, ORAC value

## Abstract

The optimum conditions for the antioxidant extraction from maqui berry were determined using a response surface methodology. A three level D-optimal design was used to investigate the effects of three independent variables namely, solvent type (methanol, acetone and ethanol), solvent concentration and extraction time over total antioxidant capacity by using the oxygen radical absorbance capacity (ORAC) method. The D-optimal design considered 42 experiments including 10 central point replicates. A second-order polynomial model showed that more than 89% of the variation is explained with a satisfactory prediction (78%). ORAC values are higher when acetone was used as a solvent at lower concentrations, and the extraction time range studied showed no significant influence on ORAC values. The optimal conditions for antioxidant extraction obtained were 29% of acetone for 159 min under agitation. From the results obtained it can be concluded that the given predictive model describes an antioxidant extraction process from maqui berry.

## 1. Introduction

Maqui (*Aristotelia chilensis [Mol]Stuntz*) is a native South America evergreen shrub. Particular attention has been paid to polyphenols and anthocyanins content in this berry, not only for its use as natural colorant, but also for its potential beneficial effects on human health, including the use as dietary supplements in functional food products [1,2]. The antioxidant capacity of maqui berry has been outstanding due to its high ORAC value, surpassing many fruits, i.e., blueberry, strawberry, raspberry, blackberry [3], Cape gooseberry and papaya [4], pomegranate, acai and cranberry [2]. This property relates maqui consumption to health benefits such as anti-diabetic and anti-inflammatory effects and the prevention of Alzheimer’s disease [2]. Besides, berries have emerged as a rich dietary source of phenolic antioxidants and bioactive properties [5]. ORAC is currently the most relevant and most widely accepted method to determine antioxidant activity. Since the ORAC assay uses fluorescence for detection, it is ideal for the antioxidant analysis of red to blue colored anthocyanin rich extracts such as maqui berry extract [6].

Conventionally, the antioxidant compounds are extracted using a suitable solvent-followed-by-evaporation method to separate the solvent from the product. The solubility and extractability of phenolic compounds in food matrices depend on solvent polarity, the nature of the polyphenols (e.g., degree of polymerization) and chemical properties of the phenolics, the composition of the food matrix and interactions between the compound and food matrix [7] besides being influenced by several extracting factors including temperature, solvent-to-sample ratio, particle size, time, the number of repeated extractions of the sample, as well as solvent type [8,9]. For a further valid comparison of the phenolic levels in berries and their derived products, it is necessary to use the same method of extraction and a comparative methodology for polyphenols quantification as well as its antioxidant capacity. The high variability of analytical methods used to quantify phenolic content is associated with sample extraction [10]. Finding new and safe antioxidants from natural sources is of great interest for applications in natural antioxidants, functional foods, and nutraceuticals [11], in addition to the application of a quick and low-cost technology. In the case of maqui berries, there is still limited available information concerning to an optimization of their antioxidant compounds.

The response surface methodology (RSM) establishes the relationship between a measured response and a variety of factors that influence an outcome [12]. Nwabueze [13] mentioned that importance to adapt the RSM as a mathematical model for bioprocess optimization is based on determining the optimum process variable combinations that maximize or minimize that product response. Thus, it is a useful tool for process optimization that allows measuring of independent variable influence on a response variable to be represented by a mathematical model that can reproduce the behavior of these parameters with a limited number of experiments [8]. Then, RSM is less laborious and time consuming than other approaches, and today is one of the most popular optimization techniques in the field of food science. Several authors have used RSM to assess the effect on the antioxidant capacity of leaves, fruits and food [14,15,16]. In order to provide a consistent data in the experimental region it is necessary to perform an optimal number of experiments under a correct design of experiments (DoE). When there are a high number of variables that must be studied or multilevel qualitative variables are present, D-optimal design is a useful choice to be used. D-optimal design is a computer-generated DoE that sets a minimal number of experiments from a pool of theoretically possible designs. It allows handling qualitative variables with two or more levels and quantitative variables at the same time. [17]. The application of D-optimal design has been reported in different areas including food technology [18,19,20], engineering [21], kinetic chemical reactions [22] and pharmaceutical field [23].

The objective of the current study was to assess the effect of three operating conditions of extraction process, namely solvent type (methanol, ethanol and acetone), solvent concentration and extraction time, on antioxidant capacity by ORAC method of the maqui berry extracts by using D-optimal design and RSM thus, a set of optimal extraction parameters can be achieved to obtain the highest values of ORAC.

## 2. Methods

### 2.1. Materials

The following reagents were used: 2,20-azobis-2-amidinopropane (AAPH) and fluorescein sodium salt were purchased by Merck (Merck, Germany). Organic solvent for analysis from Merck. Ultra-pure water was prepared by using a PW-Ultra Water System (Heal Force, Shanghai, China).

### 2.2. Extraction Protocol

Maqui berries (*Aristotelia chilensis [Mol]Stuntz*) were obtained from neighbouring forests in the town of Mulchen, Region Bio-Bio, Chile (37°43′08″ S; 72°14′27″ W). They were washed and their leaves were separated from other edible parts. The fresh berry was fragmented using an analytical mill (IKA® A-11, Wilmington, DE, USA) and immediately frozen with liquid nitrogen (1:2, *w/v*) to avoid the oxidation of the bioactive compounds. The fraction was homogenized and frozen to be used for the extracts.

One gram of finely ground maqui samples were added to 70 mL of solvents solution at different concentrations according to previous results described by Cacace and Mazza [24] introducing some modifications. The mixtures were then vigorously shaken (145 rpm) and kept at 30 °C in a water bath incubator (Memmert, WNB 22, Schwabach, Germany) for antioxidants extraction. After the evaluated extraction time, the resultant solutions were filtered through Whatman filter paper No.1. The extracts were evaporated (Multivapor Büchi P-6, Flawil, Switzerland) at 38 °C and redissolved in 10 mL methanol-formic acid (99:1). The extraction was stored at –80 °C until analyses (no longer than 48 h).

### 2.3. Determination of Antioxidant Capacity by ORAC Method

The ORAC (Oxygen radical absorbance capacity) assay was performed following the procedure described by Zhang et al. [25]. 200 µL of a fluorescein solution freshly prepared (100 nM, phosphate buffer, pH 7.4) was added to each well black plate and 40 µL of extract solution or Trolox standard solution and then incubated at 37 °C for 20 min. The assay was initiated by adding the peroxyl radical generator AAPH (2,2’-Azobis[2-amidinopropane] dihydrochoride), prepared in phosphate buffer. Specifically, 35 µL of AAPH, 0.36 M were added and the fluorescence was read (λ_ex_ = 485 nm and λ_em_ = 535 nm) every minute by using a Multilabel Plate Reader (Perkin–Elmer, Victor χ^3^, Hamburg, Germany) maintained at 37 °C and the fluorescein loss was followed until the reading declined to over 95% of the initial reading. Standards and solution extracts were run in sextuple. Results for ORAC were determined by using the differences of areas under the fluorescein kinetic decay curve. The ORAC value of each solution extract was expressed in μmol Trolox Equivalents/100 g sample (µmol TE/100 g).

### 2.4. Design of Experiment and Statistical Analysis

Based on the results of preliminary experiments, a three-level D-optimal design was conducted in this optimization study. Type of solvent (*x*_1_), concentration solvent (% *v/v*, *x*_2_) and extraction time (min, *x*_3_) were the independent variables selected to be optimized for the extraction of antioxidant compounds of maqui berry (see Table 1). ORAC value (*y*) was selected as a dependent variable. A quadratic model was selected yielding an optimization design, whose statistical evaluation is described in Table 2. A second-order interaction model can represent the system by using the quadratic polynomial shown in Equation (1) that describes the impact of the three variables on the response.

(1) $$y=\overset{k}{\underset{i=1}{\sum }}b_{0}+\overset{k}{\underset{i=1}{\sum }}b_{i}x_{i}+\overset{k}{\underset{i=1}{\sum }}b_{ii}x_{i}^{2}+\overset{k}{\underset{j=i+1}{\sum }}b_{ij}x_{i}x_{j}+\varepsilon$$

where *y* is the measured response, *b*_0_ is the constant term, *b*_i_ are the unknown regression coefficients of the variables *x*_i_ that must be determined, *b*_ii_ are the quadratic regression coefficients, *b*_ij_ are the interaction coefficients, and *ε* are the residuals. The G-efficiency parameter (Table 2) compares the performance of the D-optimal design to that of a fractional factorial design and being its 100% implies that the model is equal to a fractional factorial design. It is recommended a G-efficiency over 60%–70% [26].

The selected D-optimal design consisted of 42 experiments given in Table 3 that includes a central point of 10 replicates used for variance calculation. ANOVA was performed for statistical validation of the regression at the 95% confidence level. Optimal variable conditions that maximize the response were calculated by RSM using the SIMPLEX method. MODDE 11^®^ software (MKS Instruments AB, Umea, Sweden) was employed for ANOVA test, D-optimal design construction and RSM calculations.

## 3. Results and Discussion

### 3.1. Model Evaluation

The effect of the three independent variables (type of solvent (*x*_1_), concentration solvent (% (*v/v*), *x*_2_) and extraction time (min, *x*_3_)) on ORAC value (*y*) was investigated. Response values were power transformed (*y*^2^) to achieve a fairly normal distribution of the data, thus improving its efficiency in the ANOVA analysis and also enhances the validity of the model. A quadratic polynomial model explains significantly (*p* < 0.05) maqui antioxidant capacity (ORAC value) regarding to the variables being studied. It was found that the ORAC value varied in wide ranges 3419.5–19,105.7 μmol TE/100 g depending on the changes of process variables. Gironés-Vilaplana et al. [4] reported highest value results within this range in Chilean maqui.

**Figure 1:**
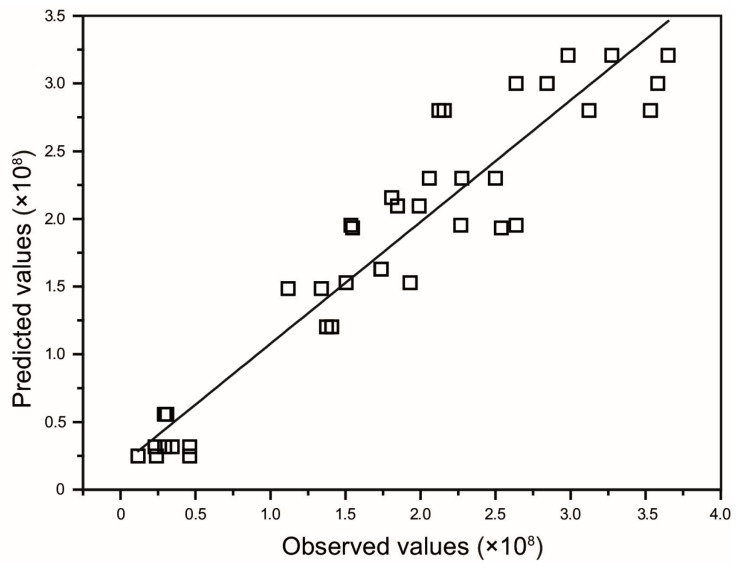
Plot of observed values vs. predicted values. Response values are power transformed *y*^2^. *R*^2^ = 0.8992.

The parameters that evaluate the selected model are described in Table 4. The goodness of fitted model was checked by the coefficient of determination (*R*^2^) which resulted 0.8992 indicating that the obtained model can explain 89.92% of variation in the antioxidant capacity value. It also shows a good prediction, where a goodness of prediction *Q*^2^ = 0.7801 was obtained. Therefore, the model is sufficiently satisfactory to explain and predict the variation in the data based on capacity antioxidant of maqui berry (see Figure 1). The model validity value 0.5800 provides information on the lack of fit and indicates that a correct model type has been chosen. The value 0.8698 for reproducibility indicates a low pure error and a satisfactory experimental procedure represented in a small variation in the replicates [17]. The ANOVA for antioxidant capacity response *y* indicates that the significance of the regression model is satisfactory (*p* ≤ 0.05) for this response. Table 5 shows that *F-*value was 22.706 and *p*-value of 0.000 imply that the model is significant. The insignificance (*p* > 0.05) on lack of fit for the model implies that this regression model is acceptable.

### 3.2. Model Interpretation

**Figure 2:**
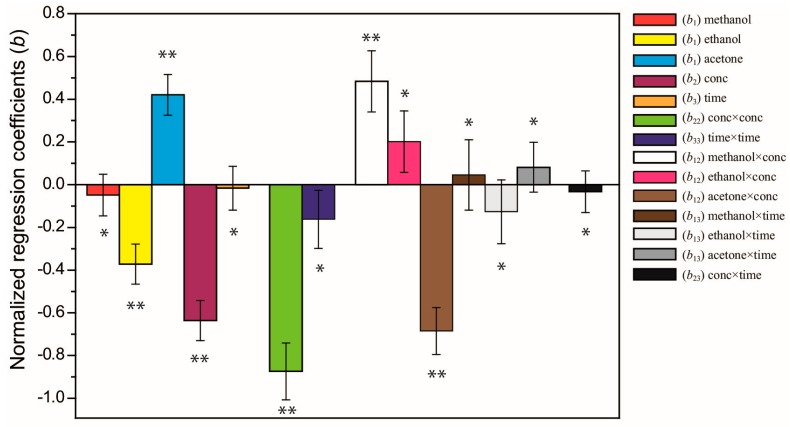
Coefficients plot of the model. The significance of the coefficients are (*) *p* > 0.05 and (**) *p* < 0.05.

Regression coefficients of the obtained model are presented as a graphical representation in Figure 2. The *p*-values were used as a tool to check the significance of each coefficient. Six significant terms have a large and significant (*p* < 0.05) effect on antioxidant capacity: two linear terms of solvent type (*b*_1_, ethanol, acetone) and solvent concentration (*b*_2_), the quadratic term of concentration×concentration (*b*_22_), and the interaction terms of methanol×concentration and acetone×concentration (*b*_12_). The other model terms, however, showed a non-significant effect (*p* > 0.05). The polynomial quadratic model for antioxidant capacity is given in Equation (2):

*y* = *b*_0_ + *b*_1_*x*_1_ + *b*_2_*x*_2_ + *b*_3_*x*_3_ + *b*_12_*x*_1_*x*_2_ + *b*_13_*x*_1_*x*_3_ + *b*_23_*x*_2_*x*_3_ + *b*_22_*x*_2_*x*_2_ + *b*_33_*x*_3_*x*_3_ (2)

**Figure 3:**
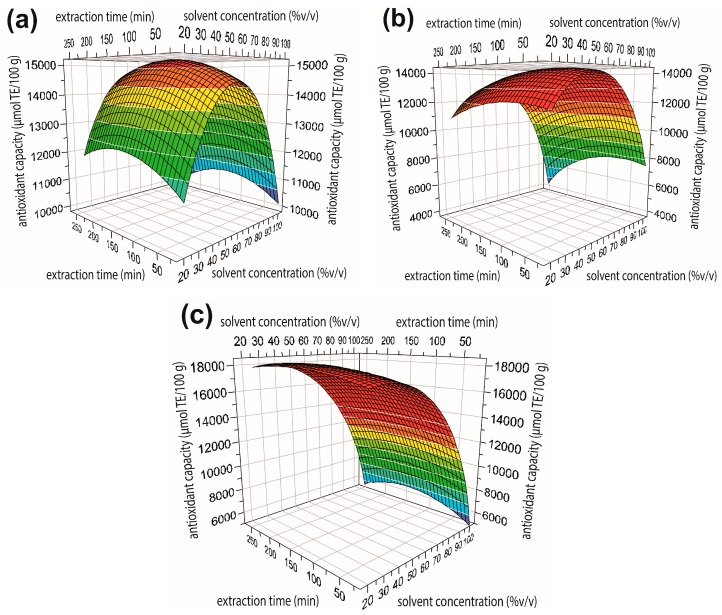
Response surface maps plotting *x*_2_ solvent concentration vs. *x*_3_ extraction time, with (**a**) *x*_1_ methanol; (**b**) *x*_1_ ethanol; and (**c**) *x*_1_ acetone.

The large and negative quadratic regression coefficient *b*_22_ for concentration (green bar in Figure 2) indicates a strong curvature in the response for the antioxidant capacity (Figure 3), in which a maximum ORAC value can be reached within the experimental region. Regarding to solvent type, it is observed that only acetone has a positive effect in ORAC value (light blue bar in Figure 2). Thus, antioxidant compounds appear to be more extractable in acetone than in methanol or ethanol. Céspedes et al. [5] indicated that in maqui extract the acetone partition showed an excellent antioxidant activity.

It is known that the concentration of a solvent is an element affecting its polarity. In a sequence from the highest polarity to the lowest are: methanol, acetone and ethanol. In this way, a higher polarity compounds are responsible for maqui berry antioxidant capacity. The methanol proved to be a better solvent for the antioxidant capacity (DPPH) in strawberry [16] likewise this solvent is used for maqui ORAC value extraction by Gironés-Vilaplana et al. [4] for anthocyanin extraction and phenols from maqui by Brauch et al. [2], Rojo et al. [27] and Escribano-Bailón et al. [1]. Due to the higher polarity of methanol, more polar phenolic compounds may be extracted from maqui berry, such as several glycosides of anthocyanidins (delphinidin, cyanidin), flavonoids (quercetin, rutin, myricetin, catechin and epi-catechin) and phenolic acids (gentisic acid, ferulic acid, gallic acid, p-coumaric acid, sinapic acid, 4-hydroxybenzoic acid) [5]. 

As the acetone concentration in aqueous solution decreases, the solvent polarity increases. Therefore, it could be seen that a higher scavenging was attained by using more methanol concentration and acetone in aqueous solution. These results were in accordance with berries studies by Arancibia et al. [28] where results indicated that the bioactive compounds (polyphenols, flavonoids, flavanols, tannins and ascorbic acid) are significantly higher in water and significantly lower in non-polar solvent.

Maqui berry has a particularly high concentration of anthocyanins, and a prominent feature of the anthocyanin composition lies on its biosynthetic pathway that is largely channeled towards the formation of polyglycosylated derivatives that are highly polar and water-soluble. These characteristics are attractive for extraction and potential use as food colorants, as well as for pharmacological uses [1].

In spite of constant extraction temperature, it is possible that this variable causes an increase of antioxidant compound solubility and thus, the liquid solvent diffusion coefficient in the solid matrix favors the desorption kinetics of matrix compounds [24]. However, high temperature has a strong influence on deployment of anthocyanins [29] and degradation of some phenolic glycosides and flavonols at higher temperatures [8]. In this experiment, the variable “temperature” was kept constant at 30 °C, so as not affect these bioactive compounds.

It is known that the extraction time is an important parameter in the extraction procedure. According to the many authors, the extraction time can either be as short as a few minutes or very long, up to 24 h. In this study, the range of extraction time was established based on practical and economical aspects (15–240 min). Heras et al. [30] mentioned that in anthocyanin extraction (main antioxidant compounds in berries) after an over prolonged time it could not be significant to pigment extraction. The Fick’s diffusion law predicted that after certain time there will be a final equilibrium between the matrix solutes and the extraction solvent. The coefficient plot indicates that the extraction time range used in this experiment showed a non-significant impact on ORAC values (light brown bar in Figure 2).

By considering the regression coefficients obtained for independent variables, solvent type and solvent concentration were the most important factors that may significantly influence ORAC value. Similarly, Liyana-Pathirana & Shahidi [31] reported that “solvent concentration” was the most important factor contributing to the extraction of phenolic compounds from wheat; and it was the “solvent” the one that had an influence on antioxidants from Borage leaves according Segovia et al. [8]. The large and negative interaction term (*b*_12_) of acetone×concentraction (brown bar in Figure 2) indicates that when acetone in used as solvent, the ORAC value increases when only a low concentration of solvent is used. However, a decreasing in ORAC values is obtained when acetone is used at high concentrations. The large and positive interaction term (*b*_12_) of methanol×concentraction (white bar in Figure 2) indicates that when methanol in used as solvent, the ORAC value increases when a high concentration of solvent is used and decreases when low concentrations are used. However, this interaction term is smaller than acetone×concentraction in magnitude. Thus, an optimal solvent and its concentration promote the extraction of specific substances of maqui berry and have a significant impact on the estimation of antioxidant capacity.

The ORAC value of maqui berry was optimized through RSM approach with SIMPLEX method. To visualize the overall effect of independent variables on ORAC value, 3D response surface maps of the quadratic polynomial were generated by varying two of the independent variables and keeping the solvent type constant as shown in Figure 3. These maps illustrate how ORAC behaves along with the variation of the percentage of solvent concentration scavenged (20%–100% (*v/v*)) and the variation of extraction time (15–240 min) regarding to each solvent. In the case of acetone (Figure 3c), it was observed that using lower acetone concentrations, the higher the ORAC value, thus from range 20%–50% (*v/v*) acetone a higher ORAC was observed, independent of the extraction time used.

In the case of ethanol (Figure 3b) it is shown that in a range of 40%–60% (*v/v*) the ORAC value is maximized. Figure 3a shows that the range of 40%–80% (*v/v*) methanol results in the highest ORAC values for this solvent. Along all extraction time range studied, no significant influence on the ORAC value for the three solvents is observed. Therefore, it could be backed up that extraction treatment at high temperatures and prolonged time causes a decrease on the antioxidant capacity according to Heras et al. [30], due to the long extraction process period that could be give rise to phenolic compounds oxidation because of the light, the exposition to oxygen and degradation. It seems that extraction time in berries of smaller diameter (4–6 mm approx.) does not significantly influence the antioxidant extraction, and therefore an adequate use of factor “time” would help to optimize the energy on the extracting processes.

Based on data collected from the experiment, the ORAC value was subjected to an optimization study with the aim of determining the optimum condition for the extraction of antioxidants from maqui berry. The optimal conditions that provide a maximum ORAC value are presented in Table 6. Comparing the predicted response of ORAC value, an error lower than 1% was found respect the experimental value obtained from a sample performed with these optimum conditions.

## 4. Conclusions

A second-order polynomial model could be used to optimize extraction of phenolic compounds from maqui berry for maximizing the total antioxidant capacity (ORAC value). Aqueous acetone was found to be the most effective solvent to extract antioxidant compounds, being more efficient than methanol and ethanol. No-significant impact was found on the response for “extraction time” variable. The optimization model showed that the extraction conditions with 29% (*v/v*) acetone and 159 min at 30 °C under agitation displayed the highest contents of ORAC value for fresh maqui. Statistical tools used, such as D-optimal design and RSM, allowed performing a suitable experiment to extract antioxidants from maqui berry. From the results above it can be concluded that the predictive model here described was a very well fitted antioxidant extracting process, according to the analyses of variance (ANOVA) and they can be applied to allow a fast, quantitative and maximum extraction of antioxidant compounds from maqui berry. 

## Figures and Tables

**Table 1:** Variables and experimental levels for the D-optimal design.

Qualitative Variables	Type		
*x*_1_: Type of solvent	Methanol	Ethanol	Acetone
	Level		
Quantitative variables	Low (–1)	Medium (0)	High (+1)
*x*_2_: Solvent concentration (% *v/v*)	20	60	100
*x*_3_: Extraction time (min)	15	127.5	240
*y*: Response variable	Antioxidant capacity (ORAC) µmol TE/100 g		

**Table 2:** Statistical evaluation of the selected D-optimal design.

**Candidate Set**	
Extreme vertices	60
Edge points	60
Centroids of high dimensional surface	15
Total runs	135
**D-optimal**	
Objective	Optimization
Model type	Quadratic
G-efficiency (%)	76.19
Condition number	4.88
Design runs	42

**Table 3:** D-optimal design with experimental data.

Runs	Variables			Response
	*x*_1_ Type of Solvent	*x*_2_ Solvent Concentration (% *v/v*)	*x*_3_ Extraction Time (min)	*y* Antioxidant Capacity (µmol TE/100 g)
1	methanol	20 (–1)^a^	127.5 (0)	13,893.9
2	methanol	100 (+1)	127.5 (0)	11,870.4
3	methanol	60 (0)	15 (–1)	14,112.3
4	methanol	60 (0)	240 (+1)	13,445.0
5	methanol	20 (–1)	127.5 (0)	12,267.6
6	methanol	100 (+1)	127.5 (0)	11,720.2
7	methanol	60 (0)	15 (–1)	13,595.0
8	methanol	60 (0)	240 (+1)	–^b^
9	ethanol	20 (–1)	127.5 (0)	11,574.7
10	ethanol	100 (+1)	127.5 (0)	5413.5
11	ethanol	60 (0)	15 (–1)	15,931.4
12	ethanol	60 (0)	240 (+1)	13,179.7
13	ethanol	20 (–1)	127.5 (0)	10,572.9
14	ethanol	100 (+1)	127.5 (0)	5560.0
15	ethanol	60 (0)	15 (–1)	12,438.1
16	ethanol	60 (0)	240 (+1)	13,179.7
17	acetone	20 (–1)	15 (–1)	18,920.9
18	acetone	100 (+1)	15 (–1)	4894.0
19	acetone	20 (–1)	240 (+1)	17,275.7
20	acetone	100 (+1)	240 (+1)	5869.2
21	acetone	20 (–1)	15 (–1)	16,240.9
22	acetone	100 (+1)	15 (–1)	4894.0
23	acetone	20 (–1)	240 (+1)	17,275.7
24	acetone	100 (+1)	240 (+1)	5429.6
25	acetone	20 (–1)	15 (–1)	–
26	acetone	100 (+1)	15 (–1)	3419.5
27	acetone	20 (–1)	240 (+1)	18,099.0
28	acetone	100 (+1)	240 (+1)	6796.7
29	acetone	20 (–1)	15 (–1)	16,864.7
30	acetone	100 (+1)	15 (–1)	6796.7
31	acetone	20 (–1)	240 (+1)	19,105.7
32	acetone	100 (+1)	240 (+1)	4817.3
33	methanol	60 (0)	127.5 (0)	15,083.9
34	methanol	60 (0)	127.5 (0)	15,808.7
35	methanol	60 (0)	127.5 (0)	14,346.8
36	ethanol	60 (0)	127.5 (0)	15,060.1
37	ethanol	60 (0)	127.5 (0)	12,399.5
38	ethanol	60 (0)	127.5 (0)	16,240.9
39	acetone	60 (0)	127.5 (0)	17,668.9
40	acetone	60 (0)	127.5 (0)	14,692.5
41	acetone	60 (0)	127.5 (0)	14,571.1
42	acetone	60 (0)	127.5 (0)	18,796.5

^a^ codified levels of quantitative variables in parenthesis. ^b^ outliers or missing values.

**Table 4:** Evaluation of the model.

Criteria	Value
*R*^2^	0.8992
*Q*^2^	0.7801
Model validity	0.5800
Reproducibility	0.8698
Condition number (*n* = 40)	4.10

**Table 5:** ANOVA test for the chosen model.

Source	Degrees of Freedom	Sums of Squares	Mean Squares	*F*-Value	*p*-Value	Standard Deviation
Total corrected	39	4.449 × 10^17^	1.141 × 10^16^			1.068 × 10^8^
Regression	11	4.001 × 10^17^	3.637 × 10^16^	22.706	0.000	1.907 × 10^8^
Residual	28	4.485 × 10^16^	1.602 × 10^15^			4.002 × 10^7^
Lack of fit (model error)	3	7.704 × 10^15^	2.568 × 10^15^	1.728	0.187	5.068 × 10^7^
Pure error (replicate error)	25	3.715 × 10^16^	1.486 × 10^15^			3.856 × 10^7^

**Table 6:** Optimal variable settings for maximum antioxidant capacity response.

Variables	Value
*x*_1_ Type of solvent	Acetone
*x*_2_ Solvent concentration (% *v/v*)	29.13
*x*_3_ Extraction time (min)	159.3
*y* Predicted response (µmol TE/100 g)	18,289.6
*y* Experimental response (µmol TE/100 g)	18,137.6
